# Prevalence Study and Genetic Typing of Bovine Viral Diarrhea Virus (BVDV) in Four Bovine Species in China

**DOI:** 10.1371/journal.pone.0121718

**Published:** 2015-04-07

**Authors:** Mingliang Deng, Sukun Ji, Wentao Fei, Sohail Raza, Chenfei He, Yingyu Chen, Huanchun Chen, Aizhen Guo

**Affiliations:** 1 The State Key Laboratory of Agricultural Microbiology, Huazhong Agricultural University, Wuhan 430070, China; 2 College of Veterinary Medicine, Huazhong Agricultural University, Wuhan 430070, China; 3 College of Animal Science, Huazhong Agricultural University, Wuhan,430070, China; 4 Key Laboratory of development of veterinary diagnostic products, Ministry of Agriculture, Wuhan 430070, China; Beijing Institute of Microbiology and Epidemiology, CHINA

## Abstract

To determine the nationwide status of persistent BVDV infection in different bovine species in China and compare different test methods, a total of 1379 serum samples from clinical healthy dairy cattle, beef cattle, yaks (*Bos grunniens*), and water buffalo (*Bubalus bubalis*) were collected in eight provinces of China from 2010 to 2013. The samples were analyzed using commercial antibody (Ab) and antigen (Ag) detection kits, and RT-PCR based on the 5’-UTR and Npro gene sequencing. Results showed that the overall positive rates for BVDV Ab, Ag and RT-PCR detection were 58.09% (801/1379), 1.39% (14/1010), and 22.64% (146/645), respectively, while the individual positive rates varied among regions, species, and farms. The average Ab-positive rates for dairy cattle, beef cattle, yaks, and water buffalo were 89.49% (298/333), 63.27% (248/392), 45.38% (236/520), and 14.18% (19/134), respectively, while the Ag-positive rates were 0.00% (0/116), 0.77% (3/392), 0.82% (3/368), and 5.97% (8/134), respectively, and the nucleic acid-positive rates detected by RT-PCR were 32.06% (42/131), 13.00% (26/200), 28.89% (52/180), and 19.40% (26/134), respectively. In addition, the RT-PCR products were sequenced and 124 5’-UTR sequences were obtained. Phylogenetic analysis of the 5’-UTR sequences indicated that all of the 124 BVDV-positive samples were BVDV-1 and subtyped into either BVDV-1b (33.06%), BVDV-1m (49.19%), or a new cluster, designated as BVDV-1u (17.74%). Phylogenetic analysis based on Npro sequences confirmed this novel subtype. In conclusion, this study revealed the prevalence of BVDV-1 in bovine species in China and the dominant subtypes. The high proportion of bovines with detectable viral nucleic acids in the sera, even in the presence of high Ab levels, revealed a serious threat to bovine health.

## Introduction

Bovine viral diarrhea virus (BVDV) is a single-stranded positive-sense RNA virus that is a member of the genus *Pestivirus* [1] and mainly affects cattle, resulting in fever, diarrhea, leucopenia, reduction in milk yield and reproductive problems [2], or no clinical symptoms, but immunosuppression [3]. Cows infected in the early gestational period with noncytopathic BVDV may produce persistently infected (PI) calves, which are mainly responsible for the spread of BVDV throughout herds via continuous viral shedding from all mucosal surfaces [4,5]. Therefore, identification and removal of such individuals is critical to the success of eradication campaigns [6]. In addition, BVDV is an etiological agent of bovine respiratory disease [7,8].

The BVDV genome contains a single open reading frame (ORF) encoding a polyprotein that is processed co- and post-transnationally into mature viral proteins. This ORF is flanked with 5’- and 3’- untranslated regions (UTRs). Based on the phylogenetic analysis of partial sequences from the 5’-UTR, the N-terminal autoprotease (Npro) or envelope glycoprotein (E2) region of the genome of this virus is usually divided into two distinct genetic species, namely BVDV-1 and BVDV-2. However, a third genetic species, BVDV-3, was recently reported [9], which was described as atypical BVDV [10]. BVDV-1 is further classified into 18 potential genetic subtypes, 1a–1t [11–16] and BVDV-2 into three subtypes (2a–2c) [17–19]. Originally, BVDV-2 was reported to be related to severe hemorrhagic disease, resulting in high mortality, in Canada [20], while BVDV-1 varies in virulence, as most BVDV-1 viruses only cause asymptomatic infection [21]. The high degree of antigenic and genetic diversity of BVDV causes major diagnostic and prophylactic difficulties because common diagnostic tests and vaccine production are based on viral antigens (Ags) [22]. Therefore, recognition of the variability of BVDV field strains is crucial when designing a successful control or eradication scheme at the herd level [6].

In China, the first description of BVDV infection in cattle dates back to 1980 when strain Changchun 184 was isolated from an aborted fetus. Since then, BVDV infection has been reported in beef and dairy cattle, yaks (*Bos grunniens*), water buffalo (*Bubalus bubalis*), camels, Sika deer (*Cervus nippon*), and swine in more than 20 regions of China with a high seroprevalence in all these species. Furthermore, the BVDV subtypes currently circulating in China are very diverse and include eight subtypes: 1a, 1b, 1c, 1d, 1m, 1o, 1p, and 1q [14,23–26]. However, previous studies were mainly performed in northern and western China, since these areas are historically the main regions with beef and dairy cattle production.

In the last decade, increasing demand by consumers and continuously increasing prices of beef and dairy products have promoted investments in cattle ranching in southern China. This change has led to an increasing number of cattle transported from the North and West to southern parts of China and has thereby altered the distribution of diseases, including BVDV infection. Therefore, nationwide re-evaluation of BVDV prevalence in bovines is important to control BVDV infection. Second, among bovine species, surveillance of BVDV infection in water buffalo and yaks less frequent compared to that in dairy cattle. Determination of the prevalence of BVDV in these species is critical to control infection. Third, in previous reports, various surveillance methods have been used by different investigators, thus the results are not comparable. Therefore, it is necessary to compare available methods in parallel to recommend the most appropriate to determine the prevalence of BVDV infection. Finally, different surveillance methods based on varied mechanisms were conducted in parallel, thus these results are expected to help determine the true prevalence of BVDV infection by evaluating the prevalence of antibodies (Abs), antigens (Ags), and nucleic acids. The aim of this study was to facilitate an evidence-based BVDV control strategy in China.

## Materials and Methods

### Sample collection

**Fig 1:**
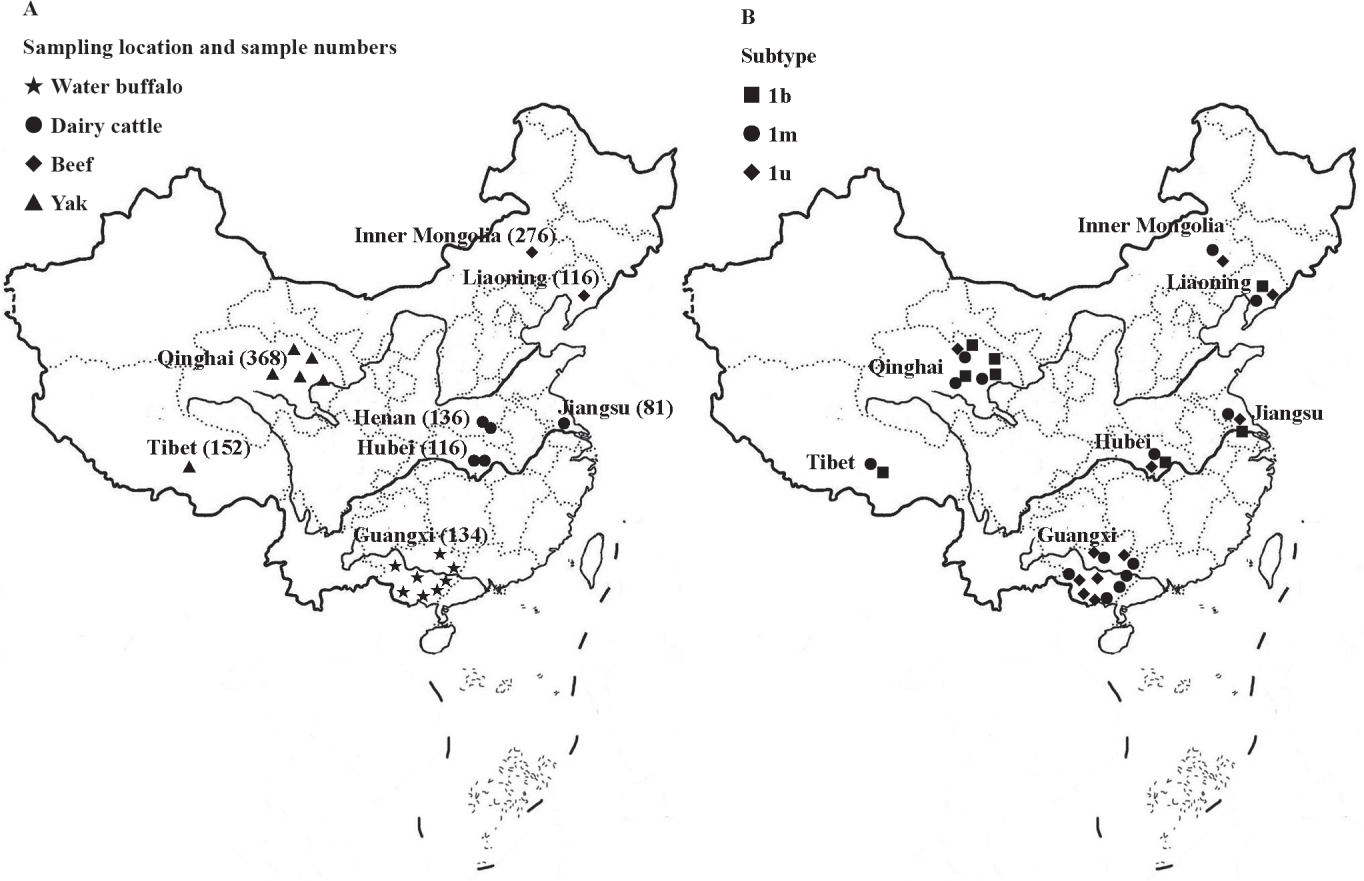
Geographic distribution of the samples and BVDV-1 subtypes. (A) The geographic locations of the samples that were collected from eight provinces of China are shown. The numbers in the brackets represent the number of samples collected from a defined region, and the symbols represent the bovine species. (B) BVDV-1 subtype distribution in the sampling areas. The symbols represent different subtypes.

This study was performed in strict accordance with the Hubei Regulations for the Administration of Affairs Concerning Experimental Animals, 2005. The study protocol was approved by the China Hubei Province Science and Technology Department (permit no: SYX-K(ER) 2010–0029). A total of 1379 serum samples were collected from eight provinces located in different regions of China from 2010 to 2013 (Table 1 and Fig 1A) which included Guangxi province in southern China, Inner Mongolia and Liaoning provinces in northern China, Qinghai province and Tibet in western China, Jiangsu province in eastern China, and Hubei and Henan provinces in central China. The species included dairy cattle, beef cattle, yaks, and water buffalo, which varied according to the primary species of the local cattle industry. The animals ranged from 2–5 years old and were clinically healthy and exhibited no reproductive problems. The herds were not vaccinated against BVDV because no commercial BVDV vaccines are available and no control programs are currently implemented in China. However, the yaks were more likely to be vaccinated with a live vaccine against classical swine fever virus (CSFV) to prevent BVDV infection due to the high incidence of BVD. Unfortunately, details regarding this heterogenic vaccination schemes were not available because the use of live CSFV vaccines is unauthorized in China. Water buffalo in Guangxi province and yaks in Qinghai province are commonly farmed species, but the herd size is usually small and the animals are free-range. Samples were collected by the local Veterinary Service Agencies for disease surveillance. In northern China, samples from beef cattle were consecutively collected at local slaughter houses for 6–9 days to ensure a sufficient sample number and diversity. In eastern and central China, samples were collected from 10% of adult cattle from one or two representative dairy farms.

Blood was collected from the jugular or caudal vein of each animal and serum was isolated, transported under cool condition to our laboratory, and stored at -20°C until assayed.

**Table 1:** Geographical distribution of serum samples.

Locations	Provinces	Cities or counties	Herd no	Collection year	Species	Sample no
Southern China	Guangxi	Liuzhou	1	2013	Water buffalo	15
		Hezhou	1	2013	Water buffalo	14
		Yulin	1	2013	Water buffalo	25
		Chongzuo	1	2013	Water buffalo	15
		Wuzhou	1	2013	Water buffalo	10
		Beihai	1	2013	Water buffalo	25
		Baise	1	2013	Water buffalo	10
		Nanning	1	2013	Water buffalo	20
Central China	Henan	Zhengzhou	2	2012	Dairy cattle	136
	Hubei	Huanggang	2	2011	Dairy cattle	116
Northern China	Inner Mongolia	Tongliao	/	2012	Beef	276
	Liaoning	Dalian	/	2012	Beef	116
Western China	Qinghai	Qilian		2012	Yak	184
		Tianjun		2012	Yak	51
		Huangyuan		2012	Yak	45
		Haiyan		2012	Yak	48
		Menyuan		2012	Yak	40
	Tibet	Lhasa		2010	Yak	152
Eastern China	Jiangsu	Nanjing	1	2010	Dairy cattle	81
	Total					1379

### Serological and antigen detection

A total of 1379 serum samples were collected and initially screened for Abs to BVDV using a commercial BVDV Ab test kit (IDEXX Laboratories, Inc., Liebefeld, Switzerland). Then, 1010 of these samples were subjected to Ag detection using the IDEXX SNAP BVDV Antigen Test kit (IDEXX Laboratories, Inc.) according to the manufacturer’s recommendations. Ag detection of the other samples was not performed because the volumes were insufficient after the first round of testing.

### Nested RT-PCR analysis

Serum samples were further divided into three groups according to the results of Ab detection, namely Ab-positive, Ab-negative and suspicious groups, and samples from each group were selected for RT-PCR analysis (S1 Table).

### Primer selection

For BVDV-1 identification, a region specific to the BVDV-1 NADL 5’-UTR (GenBank accession no. M31182) was amplified by nested RT-PCR using BVDV-1 F and BVDV-1 R as the outer primers, and BVDV-1 Fn and BVDV-1Rn as the inner primers [24].

BVDV-1-negative samples were subjected to BVDV-2 detection using outer primers (BVDV F1/R1) and inner primers (BVDV P3/P4) specific to the 5’-UTR of BVDV-2 reference strain 890 (GenBank accession no. U18059) [23].

To confirm the typing results based on 5’-UTR sequences, a 411-bp product containing the Npro region was amplified by nested RT-PCR using the outer primers B32/B31 and inner primers BD1/BD3 [13,27]. For verification of newly identified subtype isolates, the outer primers B32/B31 and inner primers BD1u/BD3u were used. The primers BD1u/BD3u were specific to BVDV-1 strain M31182 isolated from a yak in Sichuan, China (GenBank accession no. JQ799141).

All primers used in this study are listed in S2 Table.

### RNA isolation and cDNA synthesis

Total RNA was extracted from 140 μL of serum using the TIANamp virus RNA kit (Tiangen Biotech (Beijing) Co., Ltd., Beijing, China) according to the manufacturer’s instructions and stored at -70°C until assayed. Reverse transcription (RT) was performed to produce cDNA using the reagents of the PrimeScript RT reagent kit with gDNA Eraser (Takara, Otsu, Shiga, Japan) following the manufacturer’s protocol.

### Nested PCR of cDNA and sequencing

For BVDV-1 detection, primary PCR was performed using a total volume of 25 μL containing 12.5 μL of PCR Mix (Dongsheng Biotech, Guangzhou, China), 2 μL of cDNA, 9.5 μL of sterilized H_2_O, and 0.5 mM each of the primers BVDV-1 F and BVDV-1 R. The reaction was carried out at 94°C for 5 min, followed by 35 cycles of 94°C for 30 s, 56°C for 30 s, and 72°C for 30 s, with a final elongation step of 72°C for 10 min. A 2 μL aliquot from the primary PCR was used as a template for the second PCR and the reagents and cycling conditions for the secondary PCR were the same as those for the primary PCR except 30 cycles were used.

For Npro gene amplification, the reaction mixtures of primary and secondary PCR were the same as those described above. The primary PCR was carried out at 94°C for 4 min, followed by 35 cycles of 94°C for 1 min, 50°C for 30 s, and 72°C for 40 s, with a final elongation step of 72°C for 10 min, while the secondary PCR was similar except the annealing temperature was increased to 58°C.For BVDV-2 detection, the reaction mixture was the same as described above. The primary PCR reaction was included 30 cycles of 95°C for 30 s, 55°C for 30 s, and 72°C for 30 s with a final elongation step of 72°C for 10 min. The nested amplification conditions were the same as those for the primary PCR reaction, except that the annealing temperature was 53°C. Two BVDV Ag-positive samples (NMG313-1 and NMG314-65) detected by the IDEXX SNAP BVDV Antigen Test kit in this study were used as positive controls and commercial fetal bovine serum (Gibco, Grand Island, NY, USA), which was confirmed with the IDEXX SNAP BVDV Antigen Test kit and RT-PCR, was used as a negative control. The PCR-amplified amplicons were checked by electrophoresis on 1% agarose gel with EB stain, purified using the TIANgel Midi Purification Kit (Tiangen Biotech (Beijing) Co., Ltd.) and sequenced by Shanghai Sangon Biological Engineering Technology & Services Co., Ltd. (Shanghai, China) using the primers listed in S2 Table with an ABI automated 3730 sequencer (Applied Biosystems, Foster City, CA, USA). Nucleotide sequences were aligned using SeqMan II sequence assembly and analysis software (DNASTAR, Inc., Madison, WI, USA). Similarities in nucleotide sequences were evaluated using the MegAlign program (DNASTAR, Inc.) and the specified sequences against existing sequences were further identified using the Basic Local Alignment Search Tool (http://www.ncbi.nlm.nih.gov/blast/Blast.cgi).

### Phylogenetic analysis

Nucleotide sequences of the BVDV-1 5’-UTR (200 bp) and Npro gene (411 bp) fragments were aligned using the Clustal W program (www.clustal.org/). Further phylogenetic analysis was performed with the neighbor-joining method using the MEGA5 program [28,29], and evolutionary distances were calculated using the Kimura 2-parameter method. The robustness of the phylogenetic analysis and the significance of branch order were determined using the bootstrapping method based on 1000 replicates.

A total of 37 reference sequences of known BVDV-1 and BVDV-2 strains were retrieved from the NCBI GenBank database (http://www.ncbi.nlm.nih.gov/genbank). Sequences of BVDV isolates in this study were deposited in the GenBank database under the accession numbers KJ578795–KJ578918 (S3 Table). The nucleotide sequence of the Npro gene was analyzed using the same methods and parameters. The Npro gene sequences were submitted to the GenBank database under the accession numbers KP126233–KP126243.

### Statistical analysis

Prevalence was defined as the proportion of positive animals that were tested. The chi-squared test was used to analyze differences in prevalence between two groups and a probability (*p*) value < 0.05 was considered statistically significant (*) and *p* < 0.01 as very significant (**).

## Results

### Seroprevalence of BVDV infection

The overall seroprevalence of BVDV antibodies for all four bovine species was 58.09% (801/1379) (95% confidence interval (CI): 55.4%–60.7%). However, this seroprevalence varied greatly among the provinces, ranging from 14.18% to 98.53% (Table 2). Among the tested animals, dairy cattle from Henan province had the highest seroprevalence of 98.53% (95% CI: 94.8%–99.8%), and dairy cattle from Jiangsu province ranked second with a seroprevalence of 93.83% (95% CI: 86.2%–98.0%), while water buffalo from Guangxi province had the lowest seroprevalence at 14.18% (95% CI: 8.8%–21.3%). Among these species, the seroprevalence in decreasing order was as follows: dairy cattle, 89.49% (95% CI: 85.7%–92.6%); beef cattle, 63.27% (95% CI: 58.3%–68.0%); yaks, 45.38% (95% CI: 41.0%–49.8%); and water buffalo, 14.18% (95% CI: 8.8%–21.3%). Difference among all species was statistically significant (*p* < 0.001) (Table 3).

**Table 2:** Detection of BVDV infection of various cattle species in different locations.

					Ag detection	RT-PCR
Location in China	Provinces	Species	Sample no	Positive rate for Ab detection %	Positive rate for Ag detection%	Positive rate for RT-PCR detection%
South	Guangxi	Water buffalo	134	14.18(19/134)	5.97(8/134)	19.40(26/134)
Central	Henan	Dairy cattle	136	98.53(134/136)	/	/
	Hubei	Dairy cattle	116	75.86(88/116)	0.00(0/116)	52.00(26/50)
North	Inner Mongolia	Beef	276	54.35(150/276)	0.72(2/276)	15.00(15/100)
	Liaoning	Beef	116	84.48(98/116)	0.86(1/116)	11.00(11/100)
West	Qinghai	Yak	368	51.36(189/368)	0.82(3/368)	30.00(36/120)
	Tibet	Yak	152	30.92(47/152)	/	26.67(16/60)
East	Jiangsu	Dairy cattle	81	93.83(76/81)	/	16.00(16/81)
	Total		1379	58.09(801/1379)	1.39(14/1010)	22.64(146/645)

Note: “/” indicates no detection because no samples were left after Ab detection.

**Table 3:** Comparison of seropositive rate of BVDV among different species.

Species	Positive rate for Ab detection %	Positive rate for Ag detection %	Positive rate for RT-PCr detection %
Dairy cattle	89.49(298/333)^A^	0.00(0/116)^a^	32.06(42/131)^a^
Beef cattle	63.27(248/392)^B^	0.77(3/392) ^ab^	13.00(26/200)^B^
Yak	45.38(236/520)^C^	0.82(3/368) ^abc^	28.89(52/180) ^aC^
Water buffalo	14.18(19/134)^D^	5.97(8/134)^D^	19.40(26/134) ^BCd^
Total	58.09(801/1379)	1.39(14/1010)	22.64(146/645)

Note: The letters at the upper right corner indicate difference between groups by the chi-squares test. The different letters represent significant difference where upper case letters, or upper case and low case letters mean P < 0.01, while different low case letters mean P < 0.05); and the same letters represent no difference (P > 0.05).

### BVDV Ag detection

A total of 1010 serum samples, which was less than the number used for Ab detection because the volume of the other 369 samples was insufficient for Ab detection, were subjected to BVDV Ag detection, which showed that only a few samples were positive with an overall rate of 1.39% (14/1010) (95% CI: 0.8%–2.3%). Contrary to the Ab profile, the positive rate of water buffalo samples was significantly highest at 5.97% (95% CI: 2.6%–11.4%) compared to that of the other species (*p* < 0.01) (Table 3), while that of dairy cattle was lowest at 0.00% for 116 samples (95% CI: 0%–3.1%). For beef cattle and yaks, the Ag-positive rates lied between these values, but were less than 1% (Table 3). Furthermore, 13 of 14 Ag-positive samples were Ab-negative (S4 Table).

### Nested RT-PCR detection of BVDV infection

A total of 645 samples were tested for a specific fragment of the 5’-UTR of BVDV-1. These samples were chosen from the 1243 sera samples left after the first round of testing for BVDV-1 antibodies covering Ab-positive, Ab-negative, and suspected samples (S1 Table). As shown in Table 4, 146 (22.6%) of the 645 serum samples were positive and the RT-PCR products were of the right size, as indicated by electrophoresis on 1% agarose gels (S1 Fig) and had correct sequences, as indicated by aligning the sequences with the published reference sequences retrieved from the GenBank database (S3 Table).

**Table 4:** Comparisons between BVDV Ab and nucleic acid detection by RT-PCR.

	Positive no for RT-PCR	Negative no for RT-PCR	Total no for RT-PCR
Positive no for Ab detection	75	280	355
Negative no for Ab detection	63	198	261
Suspected no for Ab detection	8	21	29
Total no for Ab detection	146	499	645

The overall positive rate of nucleic acid detection by RT-PCR was 22.64% (95% CI: 19.5%–26.1%), which was 2.6-fold lower than that (58.09%) of Ab detection. Dairy cattle with the highest Ab-positive rate had the highest nucleic acid-positive rate by RT-PCR. However, unlike Ab detection, the positive rates of RT-PCR did not greatly fluctuate among the species. In decreasing order, Ab detection rates were dairy cattle, 32.06% (95% CI: 24.2%–40.8%); yaks, 28.89% (95% CI: 22.4%–36.1%); water buffalo, 19.40% (95% CI: 13.1%–27.1%); and beef cattle, 13.00% (95% CI: 8.7%–18.5%). There were significant differences between beef cattle and dairy cattle (*p* < 0.001) and yaks (*p* < 0.001), and between dairy cattle and water buffalo (*p* < 0.05), while no difference existed between yaks and beef cattle, or yaks and water buffalo (*p* > 0.05) (Table 3).

A comparison between Ag-positive and RT-PCR detection showed that 9 (64.3%) of the 14 Ag-positive samples were positive for BVDV nucleic acid, while five samples, including one of three from yaks and four of eight from water buffalo, were not confirmed by RT-PCR (S4 Table). The agreement between Ab and RT-PCR detection was compared (Table 4). Of the 355 Ab-positive samples, 75 were positive by RT-PCR, yielding a ratio of 21.27% (75/355), while of the 261 Ab-negative samples, 63 were positive by RT-PCR, yielding a ratio of 24.14% (63/261). There was no statistically significant difference between these two ratios (*p* > 0.05). On the other hand, when the samples were re-grouped based on positive and negative RT-PCR results, the Ab-positive ratio was 51.37% (75/146) among RT-PCR-positive samples and 39.68% (198/499) among RT-PCR-negative samples, indicating a significant difference between these two ratios (*p* < 0.05), thus the prevalence determined by RT-PCR was positively correlated to that of the Ab test. The ratios of Ab suspected samples were 5.48% (8/146) for RT-PCR-positive samples and 4.21% (21/499) for RT-PCR-negative samples, indicating no statistically significant difference between these two ratios (*p* > 0.05) (Table 4).

**Fig 2:**
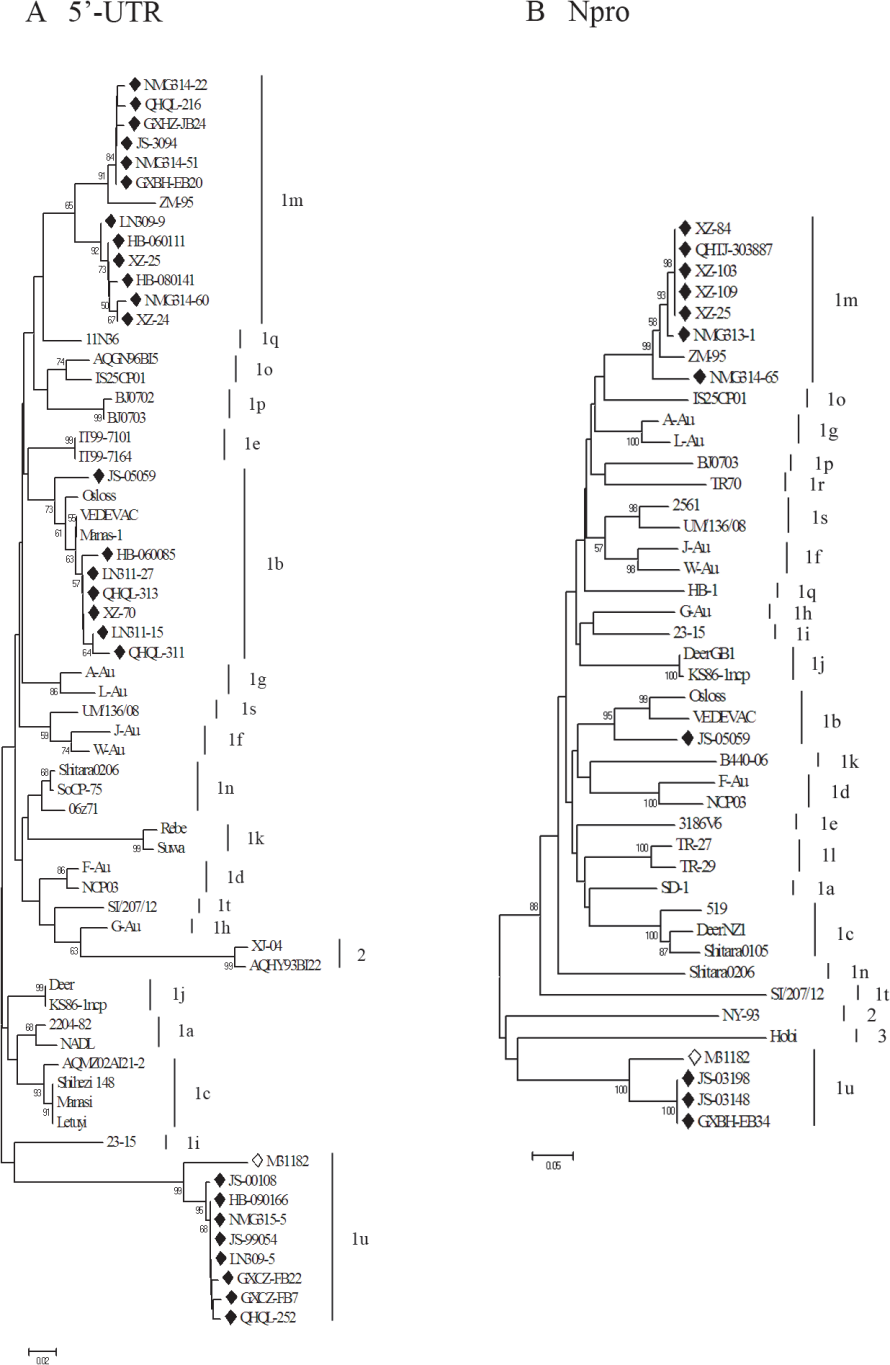
Phylogenetic analysis based on 5’-UTR (200 bp) and Npro (411 bp) sequences. A phylogenetic tree of the 5’-UTR was created using the nucleotide sequences of representative BVDV-1 isolates from each province and 37 reference strains retrieved from the GenBank database (S3 Table) (A); Phylogenetic tree analysis of the Npro gene was created using the nucleotide sequences of 11 selected BVDV-1 samples in this study and 32 BVDV reference strains retrieved from the GenBank database (B). ◆, isolates from this study; ◇, M31182 (JQ799141). The GenBank accession numbers of the reference strains used for Npro analysis were as follows: SD-1 (M96751), Osloss (M96687), VEDEVAC (AJ585412), 519 (AF144464), DeerNZ1 (U80903), Shitara0105 (AB359926), F-Au (AF287284), NCP03 (AB359927), 3186V6 (AF287282), J-Au (AF287286), W-Au (AF287290), A-Au (AF287283), L-Au (AF287287), G-Au (AF287285), 23–15 (AF287279), DeerGB1 (U80902), KS86-1ncp (AB078950), B440-06 (EU224257), TR-27 (EU163975), TR-29 (EU163977), ZM-95 (AF526381), Shitara0206 (AB359930), IS25CP01 (AB359931), BJ0703 (GU120261), HB-1 (KC695812), TR70 (KF154779), 2561 (JQ920343), UM/136/08 (LN515612), SI/207/12 (LN515611), NY-93 (AF502399), and Hobi (AY735486).

While we attempted to sequence all 146 positive samples, only 124 were successful due to technical problems, such as double signals during the sequencing process, encountered while processing the other 22 positive samples (S3 Table). All sequences belonged to BVDV-1. The BVDV-1 subtypes for each region were further analyzed (Table 5), which identified two main subtypes, namely 1b and 1m, collectively accounting for 82.26% (102/124) of all strains. Among these, 33.06% (41/124) were 1b and 49.19% (61/124) were 1m, respectively. The clinical strains classified as 1b shared a sequence homology of 94.0%–99.0% with the reference strains, while those classified as 1m shared a sequence homology of 89.7%–96.0%. The other 17.74% (22/124) BVDV-1 sequences did not match the known subtypes and clustered in a new branch, with a nucleotide homology of 97.5%–100.0%, designated as BVDV-1u. The sequences of these 22 samples were submitted to a BLAST search using the blastn algorithm. The results showed that they shared a homology of 91.5%–93.5% to the Chinese strain M31182 (GenBank accession no.: JQ799141.1) isolated from a yak in Sichuan, China (S2 Fig). However, this strain had not yet been classified into a known subtype [30] A phylogenetic tree of the representative clinical strains of each province and the reference strains (S3 Table) was constructed (Fig 2A) and the geographic distribution of the BVDV-1 subtypes was plotted (Fig 1B).

**Table 5:** BVDV-1 subtypes based on partial 5’-UTR sequences of 124 samples.

Locations in China	Provinces	Species	Subtype1b	Subtype1m	Ubtype 1u
South	Guangxi	Water buffalo	0	9	7
Central	Hubei	Dairy cattle	6	13	1
North	Inner Mongolia	Beef	0	13	2
	Liaoning	Beef	7	3	1
West	Qinghai	Yak	25	5	1
	Tibet	Yak	2	13	0
East	Jiangsu	Dairy cattle	1	5	10
Total	124		41	61	22

To confirm the typing results based on 5’-UTR sequences, Npro sequences of the 11 samples (one from group 1b, seven from group 1m and three from group 1u, the new subtype) were compared. Phylogenetic analysis was performed by comparing a 411-bp region of Npro corresponding to nt 386–796 of our 11 samples with data available for BVDV reference strains representing BVDV subtypes 1a–1r, 2, and 3. The phylogenetic analysis results confirmed the classification determined by the 5’-UTR sequences (Fig 2B). Briefly, the sample JS-05059 was classified as subtype 1b and shared a sequence homology of 87.1%–88.1% with known sequences of the same subtype; samples NMG313-1, NMG314-65, XZ-84, QHTJ-303887, XZ-103, XZ-109, and XZ-25 belonged to subtype 1m and shared a sequence homology of 93.9%–95.6% with known sequences of the same subtype; and samples JS-03198, JS-03148 and GXBH-EB34 were clustered to the 1u subtype and shared a sequence homology of 89.8% with the Chinese strain M31182 retrieved from the GenBank database. In addition, the 5’-UTR sequences of these three isolates had 93.5% identity with that of strain M31182.

## Discussion

It is difficult to determine the exact extent of the BVDV epidemic among different bovine species in China due to the variety of detection tests, sampling methods, species, and locations in individual reports. Generally speaking, most areas of China have reported the detection of BVDV [31]. For large-scale farms, the average seroprevalence rate reached 92.5% among dairy cows and 29.8% among beef cattle in Fujian province in southern China [32]. In western China, the seroprevalence of yaks was reportedly 53.65% in Tibet and 72.14% in Qinghai province [33]. The positive ratio of neutralization Ab in water buffalo averaged 17.25% in some areas [34]. These results demonstrated a high and variable seroprevalence of BVDV-1 among Chinese bovine species. Our results confirmed this status by demonstrating a seroprevalence of 89.49% (298/333) for dairy cows, 63.27% (248/392) for beef cattle, 45.38% (236/520) for yaks, and 14.18% (19/134) for water buffalo. These results are within previously reported ranges, but the seroprevalence of beef cattle was greatly increased in our study, which was probably due to the frequent movement of calves because of the rapid expansion of the beef industry. In addition, the seroprevelance among yaks in Qinghai province (51.36%) and Tibet (30.92%) in this study were lower than previously reported (72.14% and 53.65%, respectively) [33]. We propose the following reasons for these differences: (i) the samples came from different regions; and (ii) in some regions, yaks vaccinated with live CSFV vaccine might have improved the false positive rate in the cited previous reports or played a role in the control of BVDV spread leading to the later decrease in the seropositive rates in this study. According to local veterinarians, unauthorized use of live CSFV vaccine is administered to about 15%–20% of the yaks in some areas to prevent BVD outbreaks [26]. However, records for this vaccination were not available. In addition, it may be possible that some false positive results occurred because the test kit for BVDV Ab detection was validated in dairy and beef cattle, not yaks and water buffalo.

As proposed by Houe [35], the prevalence of BVDV Ab in cattle herds varies greatly and corresponds to the five phases of the infection cycle. We suspect that the herds sampled in this study might have been in phase B (infected herd with PI calves younger than 3–4 months old and most acute infections occur at varying rates, depending on housing of the animals) and phase C (an infected herd with PI calves older than 3–4 months old, in which seropositivity can reach > 90% with no eradication program).

### Correlations between Ab and Ag testing

The concept that Ab-positive rates are inversely correlated to Ag-positive rates has be confirmed in infections with BVDV [36] and other agents s [37]. The results of this study supported these previous findings. For instance, the absolute Ab-positive rates were high, while the Ag-positive rates were low, indicating infrequent reactivation of BVDV from the latent status under a background of high Ab levels. Furthermore, the decreasing order of Ab-positive rates (dairy cattle (84.49%) > beef cattle (63.27%) > yaks (45.38%) > water buffalo (14.18%)) was opposite to that of Ag-positive rates (water buffalo (5.97%) > yaks (0.82%) > beef cattle (0.77%) > dairy cattle (0%)). Accordingly, among 14 Ag-positive samples, 13 were Ab-negative (S4 Table).

The Ag-positive rate in this study might have been underestimated because, generally speaking, the sensitivity of the colloidal gold-labeled test strip is usually less than that of an enzyme-linked immunosorbent assay (ELISA). Second, according to the product data sheets, the type of sample may affect sensitivity. For example, the sensitivity of a serum sample can be 4% lower than that using tissue from a small ear notch. However, as mentioned above, the test kit for BVDV Ag detection has not been sufficiently validated in water buffalo or yaks; therefore, a higher rate of water buffalo than other species might positively influence the results. Overall, the average Ag prevalence rate of 1.4% (14/1010) for these four species in our study was close to the rates in previous reports from China and other countries [35,38].

### Correlations between Ag, Ab, and nucleic acid detection

In the present study, RT-PCR demonstrated a prevalence of 22.64% (146/645) among all bovine species, which ranged from 13.00% (26/200) for beef cattle to 32.06% (42/131) for dairy cattle. These results were in agreement with those in previous reports. For instance, in previous reports, RT-PCR revealed a prevalence of BVDV of 24% (98/407) among yaks from Qinghai province [26,33], 26.85% (105/391) among dairy cattle from Ningxia province in northwestern China [25], and 29.03% (18/62) among beef cattle from northern and Eastern China [14]. Taken altogether, the proportion of bovines with viremia in China was high, although the Ab-positive rates were high.

Reportedly, RT-PCR analysis of BVDV nucleic acids is more sensitive than Ag detection. For example, Oem et al [36]detected BVDV nucleic acid in 15.5% of brain samples by RT-PCR, while only 2.9% of positive samples were confirmed by Ag detection via immunohistochemical analysis and Ag capture ELISA. Similarly, there was a large difference in positive rates between BVDV nucleic acid and Ag detection in this study.

A second possible reason for the difference between results by RT-PCR and Ag detection could be that RT-PCR can detect the presence of virus in both acutely and persistently infected animals, while Ag detection was designed for only persistently infected animals. Therefore, not all samples deemed positive by RT-PCR can be detected using the Ag test [36].

On the other hand, among the 14 Ag-positive samples in this study, 64% (9/14) were according to RT-PCR results, although the five samples not confirmed by RT-PCR may be false positives. Coincidently, these five samples included four from water buffalo and one from a yak. This fact again aroused suspicion that the Ag-capture test might be problematic in testing yaks and water buffalo. According to the product instructions, retesting of Ag-positive samples after 3 weeks is recommended to exclude false positive samples. However, resampling and testing is sometimes difficult because the cattle might be slaughtered or sold, or the travel distance is economically unviable.

As shown in Table 4 and reported elsewhere [36], of the cattle with positive results by to RT-PCR, both Ab-positive and-negative results were detected. The double positive status to both BVDV Ab and nucleic acid detection might indicate an acute infection stage, while that positive to nucleic acid but negative to Ab detection might suggest either an early phase of acute infection, because development of detectable Ab usually requires 1–2 weeks after infection, or a persistent infection in which Ab detection is negative, but positive for both nucleic acid and Ag detection. The fact that more than 30% of dairy cows, 2–5 years, in China were viremic for BVDV, particularly since all of these animals appeared "healthy," was astonishing. However, considering the fact that there is no vaccination program or eradication plan in China, the virus will continue to persistently circulate among bovine populations without interference, thus it is not difficult to understand the high prevalence. Furthermore, the dominant genotype was BVDV-1 in this study, although this genotype usually does not cause serious clinical illness and thus infected bovines may understandably appear “healthy.” Although BVDV infection is known to cause reproductive and productive abnormalities, there was no indication of such problems among the sampled herds.

On the other hand, the status of negative to RT-PCR detection, but positive to Ab detection might represent a stage after clearance of acute infection without persistence. As previously reported, PI with BVDV in cattle only occurs during fetal development, thus the status can be maintained for long periods until the PI cattle become pregnant and give birth to PI calves [39]. These PI cattle then develop immune tolerance at the fetal stage. However, acute infection of bovines without PI can induce protective immunity and finally clear the virus after acute infection.

### Epidemic BVDV genotypes in cattle in China

A variety of BVDV-1 subtypes, including 1a, 1b, 1c, 1d, 1m, 1o, and 1q, currently circulate among susceptible animals in China, 1b accounted for 11.11% (2/18), 1m for 66.67% (12/18), and a new subtype, tentatively typed as BVDV-1p, accounted for 22.22% (4/18), was detected in 18 of 62 samples, including clinical samples of diseased cattle and clinically health animals between 2005 and 2008 [14]. Subtypes 1b (75%, 18/24) and 1c (25%, 6/24) were detected in 24 samples chosen from 202 BVDV-positive samples collected from 15 cattle farms in seven districts of the Xinjiang Uygur Autonomous Region from 2006 to 2008 [23]. Subtype 1a (5%, 1/20), 1b (30%, 6/20), 1m (30%, 6/20), 1o (5%, 1/20), and an unknown subtype, which was tentatively typed as BVDV-1q, (30%, 6/20) were detected in 20 samples chosen from 137 BVDV-positive samples collected from diseased pigs in 11 provinces in China between 2007 and 2010 [24]. In addition, 13 ncp-BVDV strains were isolated from 105 (26.9%) of 391 samples collected from five dairy farms in Ningxia, China during the 2010–2011 period and subtypes 1b (23.07%, 3/13), 1d (46.15%, 6/13), and a novel subtype, which was also typed as 1q, (30.77%, 4/13) were detected among these ncp-BVDV strains [25]. Subtypes 1b (25%, 4/16), 1d (31.25%, 5/16), and 1q, (43.75%, 7/16) were detected in 16 positive samples selected from 98 of 407 samples collected from yaks in six counties of Qinghai province between 2010 and 2012 [26]. As descripted above, subtypes 1b, 1m, and 1q are commonly considered to be the dominant BVDV1 strains circulating in Chinese bovine and porcine species.

In this study, we demonstrated that 1b (33%) and 1m (49%) were dominant BVDV subtypes, accounting for 82% (102/124) of the total. These results were in agreement with those of previous reports. BVDV-1a, 1c, 1d, 1o, 1p, and 1q were not detected in this study. Theoretically, the primers used for 5’-UTR amplification in this study could detect other subtypes, including 1a, 1c, 1o, 1p, and 1q [24]. Actually, we detected 1a, 1c, and 1p once each from clinical samples of diseased cattle during routine diagnosis using these primers. In this survey, all samples were collected from clinically healthy animals. Therefore, we suspect that other factors, such as individual herds, geographical distribution of the herds, sampling size, and health status, might affect the detection results. Regarding subtype 1q, a high prevalence was found in yaks in Qinghai province [26], dairy farms in Ningxia province [25], and pigs [24]. However, this study failed to detect this subtype for several possible reasons, including: (i) the samples were collected in northeast of Qinghai province in this study, whereas samples were collected in southeast areas of Qinghai and Ningxia provinces by Gong et al. [25,26]; (ii) more samples (n = 407) were collected from yaks than in our study (n = 407 vs. 120, respectively), thus a greater number of samples should be tested to confirm the prevalence of BVDV subtype 1q in future studies; and (iii) about 15%–20% of the yaks received live CSFV vaccine, thus and subtype 1q may have been introduced by BVDV-contaminated live CSFV vaccine, as discussed by Gong et al. [26]. To date, subtype 1o has only been detected in pigs.

In addition, this study is the first to report that the new subtype BVDV-1u comprised 18% of all the detected strains. This subtype shared a high homology in the sequences of the 5’-UTR (93.5%) and Npro (89.8%) genes with the untyped strain M31182 (GenBank accession no.: JQ799141.1), which was isolated from a yak in Sichuan province, located in central China, in 2010 [30]. Other than the one subtype 1u isolate detected from a yak in Qinghai province, we also detected this subtype in three other species from other regions, including seven samples from water buffalo in Guangxi province, ten from dairy cattle in Jiangsu province and one from Hubei province, and two from beef cattle in Inner Mongolia and one from Liaoning province, respectively. This is the first report to describe the distribution of BVDV-1u in varied bovine populations from different areas in China.

## Supporting Information

S1 Fig RT-PCR products specific to the 5’-UTR of BVDV-1 on 1% agarose gel from selected serum samples. A: M: DNA ladder DL2000; Lanes 1–20: selected samples from Liaoning province designated from lane 1 to 20 as LN309-14, LN309-21, LN309-16, LN309-23, LN309-1, LN309-9, LN309-4, LN309-12, LN309-20, LN309-25, LN309-10, LN311-6, LN311-17, LN311-8, LN311-3 LN311-28 LN311-19 LN311-18 LN311-10, and LN311-27, respectively; B: M: DNA ladder DL2000; Lanes 1–20 contained partial samples from Guangxi province, designated as GXYL-KB22, GXYL-KB25, GXYL-KB14, GXYL-KB31, GXYL-KB56, GXYL-KB13, GXYL-KB19, GXYL-KB10, GXYL-KB31, GXYL-KB29, GXYL-KB6, GXYL-KB34, GXYL-KB53, GXYL-KB4, GXCZ-FB13, GXCZ-FB29, GXCZ-FB28, GXCZ-FB7, GXCZ-FB12, and GXCZ-FB5 respectively. In both A and B, lanes 21 and 22 contained positive controls (NMG313-1 and NMG314-65), which were BVDV Ag-positive samples detected by the IDEXX SNAP BVDV Antigen Test kit; lane 23 contained a negative control (fetal bovine serum; Gibco, Grand Island, NY, USA), which was confirmed as negative by the IDEXX SNAP BVDV Antigen Test kit and RT-PCR; lane 24 contained a mock control.
(TIF)

S2 Fig The homologies between 5’-UTR sequences (49–249 nt) of the BVDV-1u isolates and the BVDV-1 M31182 strain (GenBank: JQ799141). They ranged from 91.5% to 93.5%. Information regarding each isolate is listed in S3 Table.
(TIF)

S1 Table Proportion of samples tested by RT-PCR within each antibody category. (DOCX)

S2 Table Primer sets used in this study. (DOCX)

S3 Table 5’-UTR sequences of isolates and reference strains retrieved from GenBank. (DOCX)

S4 Table Antigen positive samples were detected with RT-PCR and antibody ELISA. (DOCX)
