# Correction: Prevalence Study and Genetic Typing of Bovine Viral Diarrhea Virus (BVDV) in Four Bovine Species in China

**DOI:** 10.1371/journal.pone.0134777

**Published:** 2015-07-31

**Authors:** Mingliang Deng, Sukun Ji, Wentao Fei, Sohail Raza, Chenfei He, Yingyu Chen, Huanchun Chen, Aizhen Guo

The authors have prepared a revised manuscript to address comments left by readers regarding the species examined in this article as well as the classification of pestiviruses. Please view a revised manuscript provided as Supporting Information (S1 File), a revised Fig 2, and revised Supporting Information files S1 and S3 Tables here.

**Fig 2:**
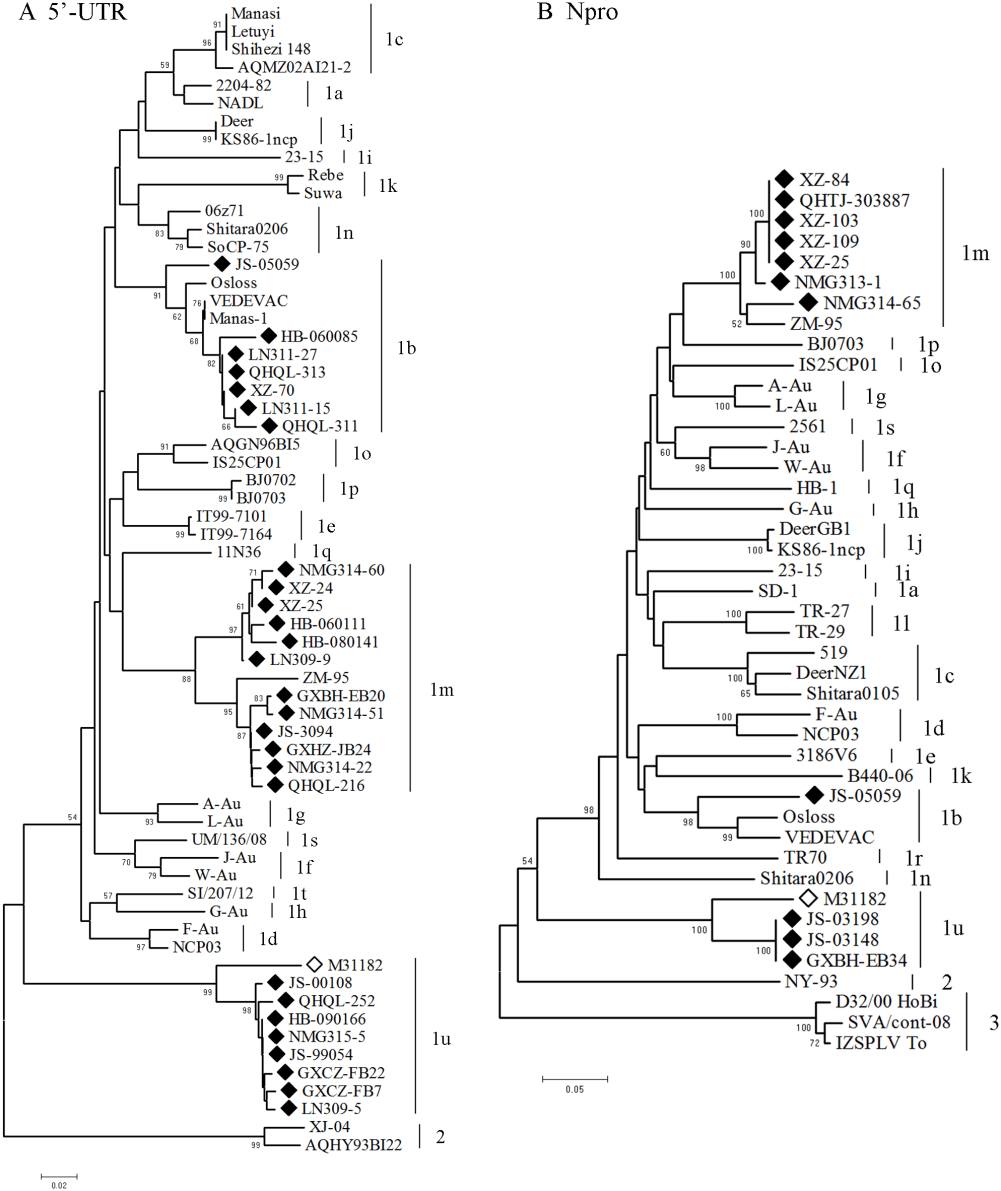
Phylogenetic analysis based on 5’-UTR (200 bp) and Npro (411 bp) sequences. A phylogenetic tree of the 5’-UTR was created using the nucleotide sequences of representative BVDV-1 isolates from each province and 37 reference strains retrieved from the GenBank database (S3 Table) (A); Phylogenetic tree analysis of the Npro gene was created using the nucleotide sequences of 11 selected BVDV-1 samples in this study and 32 BVDV reference strains retrieved from the GenBank database (B). ◆, isolates from this study; ◇, M31182 (JQ799141). The GenBank accession numbers of the reference strains used for Npro analysis were as follows: SD-1 (M96751), Osloss (M96687), VEDEVAC (AJ585412), 519 (AF144464), DeerNZ1 (U80903), Shitara0105 (AB359926), F-Au (AF287284), NCP03 (AB359927), 3186V6 (AF287282), J-Au (AF287286), W-Au (AF287290), A-Au (AF287283), L-Au (AF287287), G-Au (AF287285), 23–15 (AF287279), DeerGB1 (U80902), KS86-1ncp (AB078950), B440-06 (EU224257), TR-27 (EU163975), TR-29 (EU163977), ZM-95 (AF526381), Shitara0206 (AB359930), IS25CP01 (AB359931), BJ0703 (GU120261), HB-1 (KC695812), TR70 (KF154779), 2561 (JQ920343), UM/136/08 (LN515612), SI/207/12 (LN515611), NY-93 (AF502399), and Hobi (AY735486).

## Supporting Information

S1 File A revised version of the article. (PDF)

S1 Table Proportion of samples tested by RT-PCR within each antibody category. (DOCX)

S3 Table 5’-UTR sequences of isolates and reference strains retrieved from GenBank. (DOCX)
