# Supplementary figures and images for: Prevalence Study and Genetic Typing of Bovine Viral Diarrhea Virus (BVDV) in Four Bovine Species in China
Source: PLoS One. 2015 Apr 7;10(4):e0121718. doi: 10.1371/journal.pone.0121718 (PMC4388703; doi:10.1371/journal.pone.0121718)

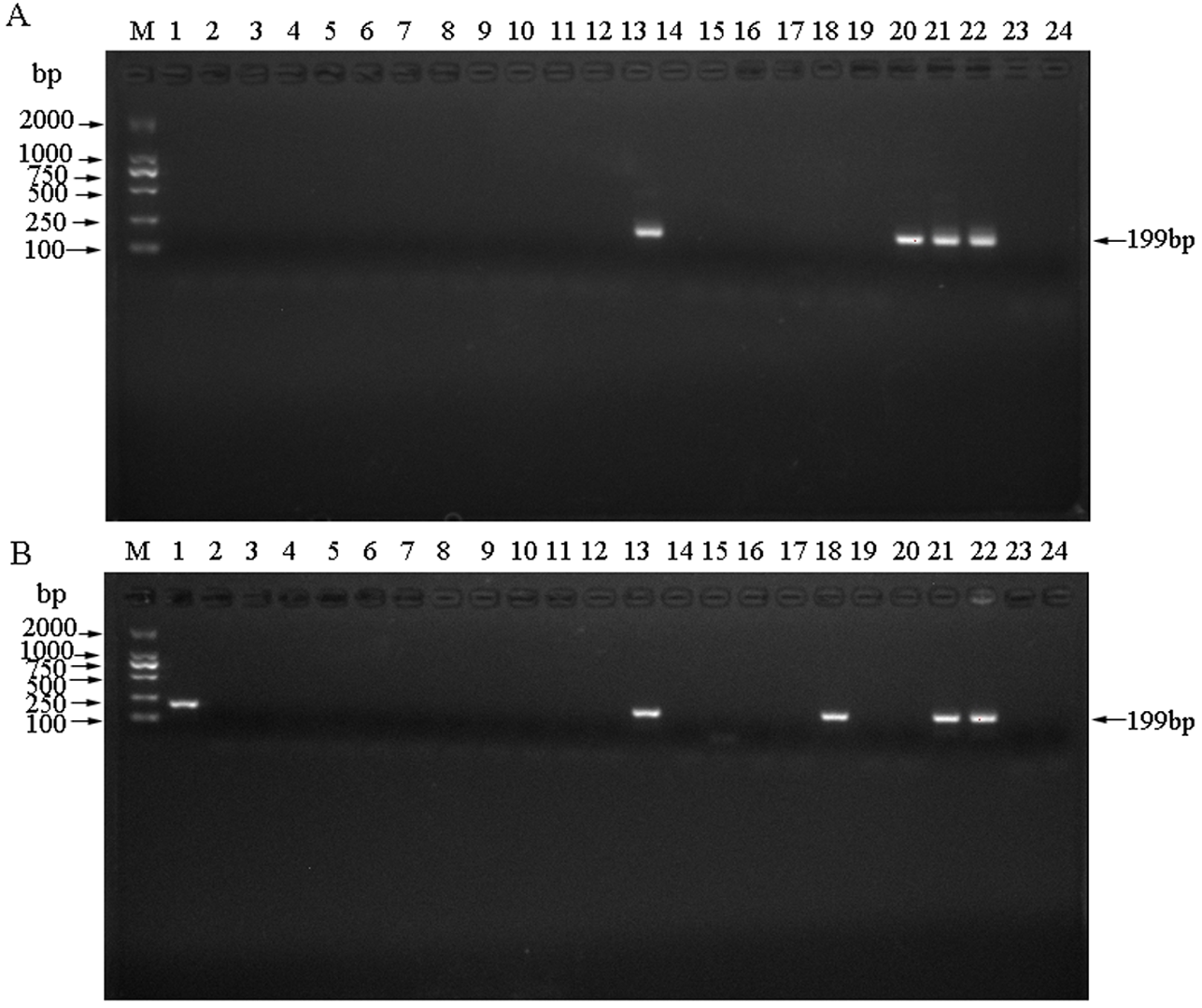

Supplement: S1 Fig — A: M: DNA ladder DL2000; Lanes 1–20: selected samples from Liaoning province designated from lane 1 to 20 as LN309-14, LN309-21, LN309-16, LN309-23, LN309-1, LN309-9, LN309-4, LN309-12, LN309-20, LN309-25, LN309-10, LN311-6, LN311-17, LN311-8, LN311-3 LN311-28 LN311-19 LN311-18 LN311-10, and LN311-27, respectively; B: M: DNA ladder DL2000; Lanes 1–20 contained partial samples from Guangxi province, designated as GXYL-KB22, GXYL-KB25, GXYL-KB14, GXYL-KB31, GXYL-KB56, GXYL-KB13, GXYL-KB19, GXYL-KB10, GXYL-KB31, GXYL-KB29, GXYL-KB6, GXYL-KB34, GXYL-KB53, GXYL-KB4, GXCZ-FB13, GXCZ-FB29, GXCZ-FB28, GXCZ-FB7, GXCZ-FB12, and GXCZ-FB5 respectively. In both A and B, lanes 21 and 22 contained positive controls (NMG313-1 and NMG314-65), which were BVDV Ag-positive samples detected by the IDEXX SNAP BVDV Antigen Test kit; lane 23 contained a negative control (fetal bovine serum; Gibco, Grand Island, NY, USA), which was confirmed as negative by the IDEXX SNAP BVDV Antigen Test kit and RT-PCR; lane 24 contained a mock control. (TIF) [file pone.0121718.s001.tif]

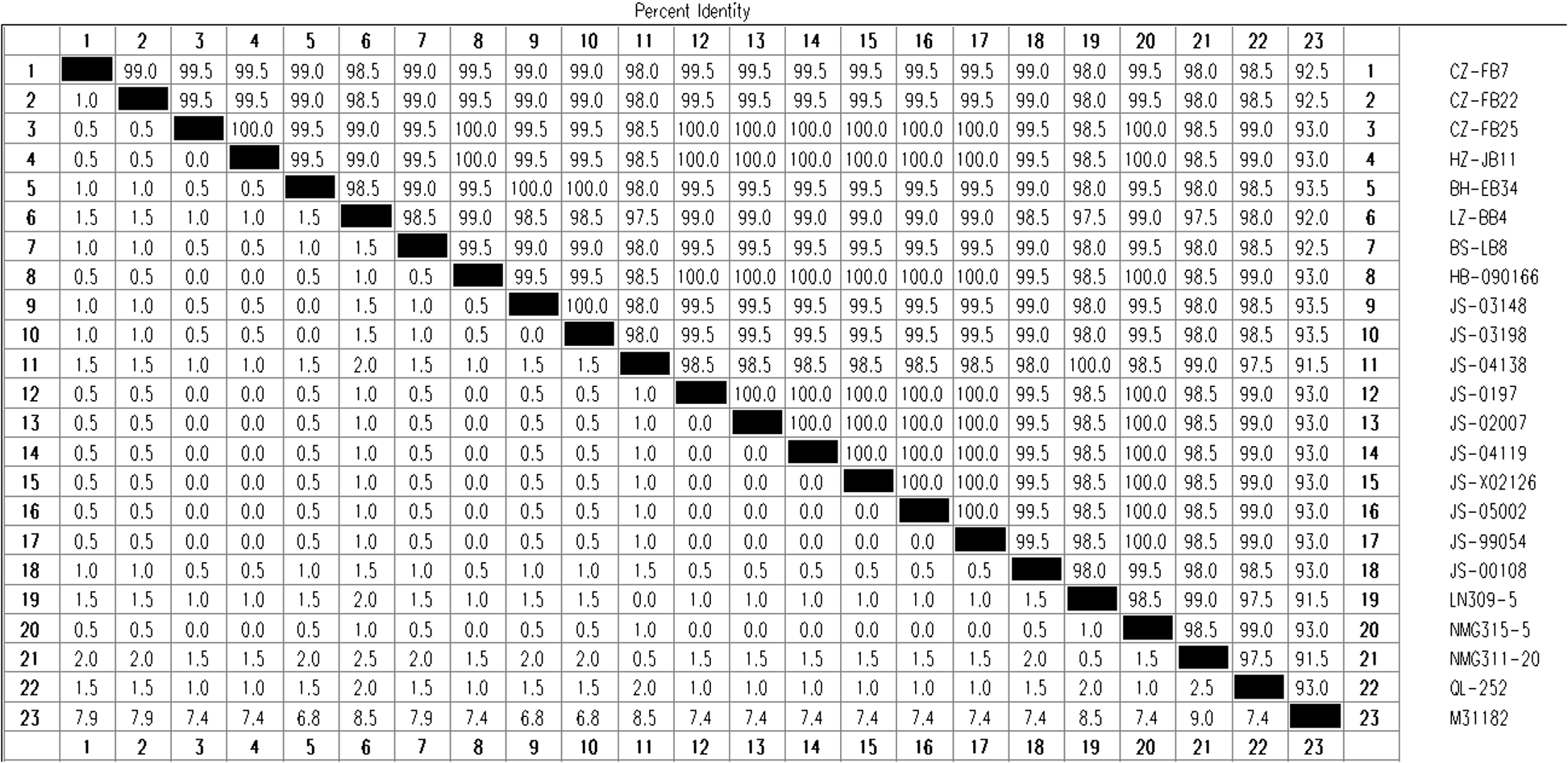

Supplement: S2 Fig — They ranged from 91.5% to 93.5%. Information regarding each isolate is listed in S3 Table. (TIF) [file pone.0121718.s002.tif]
