# Supplementary material for: Prevalence Study and Genetic Typing of Bovine Viral Diarrhea Virus (BVDV) in Four Bovine Species in China
Source: PLoS One. 2015 Apr 7;10(4):e0121718. doi: 10.1371/journal.pone.0121718 (PMC4388703; doi:10.1371/journal.pone.0121718)
Supplement: S1 Table — (DOCX) [file pone.0121718.s003.docx]

**Table S1** Proportion of samples tested by RT-PCR within each antibody category

| Locations in China | Provinces | Species | RT-PCR No/antibody positive No | RT-PCR No /antibody negative No | RT-PCR No /antibody suspected No |
| --- | --- | --- | --- | --- | --- |
| South | Guangxi | Water buffalo | 19/19 | 101/101 | 14/14 |
| Middle | Hubei | Dairy cattle | 33/88 | 15/26 | 2/2 |
| North | Inner Mongolia | Beef | 57/150 | 42/124 | 1/2 |
|  | Liaoning | Beef | 86/98 | 13/17 | 1/1 |
| West | Qinghai | Yak | 64/189 | 51/168 | 5/11 |
|  | Tibet | Yak | 20/47 | 37/100 | 3/5 |
| East | Jiangsu | Dairy cattle | 76/76 | 2/2 | 3/3 |
| Total ratio | | | 53%(355/667) | 48%(261/538) | 74%(29/38) |
