# Supplementary material for: Prevalence Study and Genetic Typing of Bovine Viral Diarrhea Virus (BVDV) in Four Bovine Species in China
Source: PLoS One. 2015 Apr 7;10(4):e0121718. doi: 10.1371/journal.pone.0121718 (PMC4388703; doi:10.1371/journal.pone.0121718)
Supplement: S2 Table — (DOCX) [file pone.0121718.s004.docx]

**S2 Table** Primer sets used in this study

| Target genes | Primers | Sequence(5’→3’) | GenBank accession no | Position in genes(nt） | Detection | References |
| --- | --- | --- | --- | --- | --- | --- |
| 5’UTR | BVDV-1 F | TCAGCGAAGGCCGAAAAGAGG | M31182 | 81-101 | BVDV-1 | [Deng et al., 2012](#_ENREF_7) |
|  | BVDV-1 R | TCCATGTGCCATGTACAGCAGAG |  | 368-390 |  |  |
|  | BVDV-1Fn | CAGTGGCGAGTTCGTTGGATG |  | 147-167 |  | [Deng et al., 2012](#_ENREF_7) |
|  | BVDV-1Rn | GGCCTCTGCAGCATCCTATCAG |  | 325-346 |  |  |
|  | BVDV F1 | AGCCATGCCCTTAGTAGGACT | M31182 | 104-124 | BVDV-2 | [Zhong et al., 2011](#_ENREF_41) |
|  | BVDV R1 | ACTCCATGTGCCATGTACA |  | 374-392 |  |  |
|  | BVDV P3 | CGACACTCCATTAGTTGAGG | U18059 | 204-223 |  | [Zhong et al., 2011](#_ENREF_41) |
|  | BVDV P4 | GTCCATAACGCCACGAATAG |  | 301-320 |  |  |
| Npro | B32 | TGCTACTAAAAATCTCTGCTGT | M31182 | 355-376 | Npro | Toplak et al., 2004 |
|  | B31 | CCATCTATRCAYACATARATGTGGT |  | 771-795 |  |  |
|  | BD1 | TCTCTGCTGTACATGGCACATG | M31182 | 367–388 |  | Vilcek et al., 2001 |
|  | BD3 | CCATCTATRCACACATAAATGTGGT |  | 771–795 |  |  |
|  | BD1u | TCTCTGCTGTACATGGCACATGGA | JQ799141 | 270-293 | Subtype 1u | This study |
|  | BD3u | CCGTCTATACACACATATATGTGGTAC |  | 672-698 |  |  |
