# Supplementary material for: Prevalence Study and Genetic Typing of Bovine Viral Diarrhea Virus (BVDV) in Four Bovine Species in China
Source: PLoS One. 2015 Apr 7;10(4):e0121718. doi: 10.1371/journal.pone.0121718 (PMC4388703; doi:10.1371/journal.pone.0121718)
Supplement: S4 Table — (DOCX) [file pone.0121718.s006.docx]

**Table S4** Antigen positive samples were detected with RT-PCR and antibody ELISA

| Antigen positive samples | Origin | Location | RT-PCR | Antibody |
| --- | --- | --- | --- | --- |
| NMG313-1 | Beef | Inner Mongolia | + | - |
| NMG314-65 | Beef | Inner Mongolia | + | - |
| LN311-27 | Beef | Liaoning | + | - |
| QHQL-336 | Yak | Qinghai | + | + |
| QHHN-48 | Yak | Qinghai | - | - |
| QHTJ-303887 | Yak | Qinghai | + | - |
| GXCZ-FB7 | Water buffalo | Guangxi | + | - |
| GXBH-EB20 | Water buffalo | Guangxi | + | - |
| GXBH-EB34 | Water buffalo | Guangxi | + | - |
| GXLZ-BB4 | Water buffalo | Guangxi | + | - |
| GXBH-EB53 | Water buffalo | Guangxi | - | - |
| GXBH-EB51 | Water buffalo | Guangxi | - | - |
| GXBH-EB39 | Water buffalo | Guangxi | - | - |
| GXNN-AB8 | Water buffalo | Guangxi | - | - |
