# Supplementary material for: Correction: Prevalence Study and Genetic Typing of Bovine Viral Diarrhea Virus (BVDV) in Four Bovine Species in China
Source: PLoS One. 2015 Jul 31;10(7):e0134777. doi: 10.1371/journal.pone.0134777 (PMC4521862; doi:10.1371/journal.pone.0134777)
Supplement: S3 Table — (DOCX) [file pone.0134777.s003.docx]

**Table S3** 5’-UTR sequences of isolates and reference strains retrieved from GenBank

| Strains | | Origin | Location | Collection year | | GenBank  accession | Subtype |
| --- | --- | --- | --- | --- | --- | --- | --- |
| **BVDV-1 isolates from this study** | | | | | | | |
| GXBH-EB20 | Water buffalo | | Guangxi | 2013 | | KJ578811 | 1m |
| GXBH-EB33 | Water buffalo | | Guangxi | 2013 | | KJ578812 | 1m |
| GXBH-EB34 | Water buffalo | | Guangxi | 2013 | | KJ578813 | 1u |
| GXBS-LB3 | Water buffalo | | Guangxi | 2013 | | KJ578818 | 1m |
| GXBS-LB8 | Water buffalo | | Guangxi | 2013 | | KJ578819 | 1u |
| GXCZ-FB22 | Water buffalo | | Guangxi | 2013 | | KJ578807 | 1u |
| GXCZ-FB25 | Water buffalo | | Guangxi | 2013 | | KJ578808 | 1u |
| GXCZ-FB7 | Water buffalo | | Guangxi | 2013 | | KJ578806 | 1u |
| GXHZ-JB11 | Water buffalo | | Guangxi | 2013 | | KJ578809 | 1u |
| GXHZ-JB24 | Water buffalo | | Guangxi | 2013 | | KJ578810 | 1m |
| GXLZ-BB13 | Water buffalo | | Guangxi | 2013 | | KJ578817 | 1m |
| GXLZ-BB4 | Water buffalo | | Guangxi | 2013 | | KJ578814 | 1u |
| GXLZ-BB5 | Water buffalo | | Guangxi | 2013 | | KJ578815 | 1m |
| GXLZ-BB6 | Water buffalo | | Guangxi | 2013 | | KJ578816 | 1m |
| GXYL-KB22 | Water buffalo | | Guangxi | 2013 | | KJ578820 | 1m |
| GXYL-KB53 | Water buffalo | | Guangxi | 2013 | | KJ578821 | 1m |
| HB-0105 | Dairy cattle | | Hubei | 2011 | | KJ578830 | 1m |
| HB-0134 | Dairy cattle | | Hubei | 2011 | | KJ578829 | 1b |
| HB-0244 | Dairy cattle | | Hubei | 2011 | | KJ578837 | 1m |
| HB-0258 | Dairy cattle | | Hubei | 2011 | | KJ578827 | 1b |
| HB-0273 | Dairy cattle | | Hubei | 2011 | | KJ578831 | 1m |
| HB-0275 | Dairy cattle | | Hubei | 2011 | | KJ578834 | 1m |
| HB-0288 | Dairy cattle | | Hubei | 2011 | | KJ578826 | 1b |
| HB-050030 | Dairy cattle | | Hubei | 2011 | | KJ578835 | 1m |
| HB-050057 | Dairy cattle | | Hubei | 2011 | | KJ578841 | 1m |
| HB-050062 | Dairy cattle | | Hubei | 2011 | | KJ578838 | 1m |
| HB-060085 | Dairy cattle | | Hubei | 2011 | | KJ578825 | 1b |
| HB-060111 | Dairy cattle | | Hubei | 2011 | | KJ578822 | 1m |
| HB-080141 | Dairy cattle | | Hubei | 2011 | | KJ578823 | 1m |
| HB-080146 | Dairy cattle | | Hubei | 2011 | | KJ578839 | 1m |
| HB-080147 | Dairy cattle | | Hubei | 2011 | | KJ578840 | 1m |
| HB-0899 | Dairy cattle | | Hubei | 2011 | | KJ578833 | 1b |
| HB-090166 | Dairy cattle | | Hubei | 2011 | | KJ578836 | 1u |
| HB-090219 | Dairy cattle | | Hubei | 2011 | | KJ578828 | 1b |
| HB-090268 | Dairy cattle | | Hubei | 2011 | | KJ578831 | 1m |
| HB-090913 | Dairy cattle | | Hubei | 2011 | | KJ578824 | 1m |
| JS-00108 | Dairy cattle | | Jiangsu | 2009 | | KJ578848 | 1u |
| JS-01159 | Dairy cattle | | Jiangsu | 2009 | | KJ578843 | 1m |
| JS-0197 | Dairy cattle | | Jiangsu | 2009 | | KJ578853 | 1u |
| JS-02007 | Dairy cattle | | Jiangsu | 2009 | | KJ578854 | 1u |
| JS-03105 | Dairy cattle | | Jiangsu | 2009 | | KJ578845 | 1m |
| JS-03140 | Dairy cattle | | Jiangsu | 2009 | | KJ578846 | 1m |
| JS-03148 | Dairy cattle | | Jiangsu | 2009 | | KJ578850 | 1u |
| JS-03198 | Dairy cattle | | Jiangsu | 2009 | | KJ578851 | 1u |
| JS-04119 | Dairy cattle | | Jiangsu | 2009 | | KJ578855 | 1u |
| JS-04138 | Dairy cattle | | Jiangsu | 2009 | | KJ578852 | 1u |
| JS-04198 | Dairy cattle | | Jiangsu | 2009 | | KJ578844 | 1m |
| JS-05002 | Dairy cattle | | Jiangsu | 2009 | | KJ578857 | 1u |
| JS-05059 | Dairy cattle | | Jiangsu | 2009 | | KJ578849 | 1b |
| JS-3094 | Dairy cattle | | Jiangsu | 2009 | | KJ578842 | 1m |
| JS-99054 | Dairy cattle | | Jiangsu | 2009 | | KJ578847 | 1u |
| JS-X02126 | Dairy cattle | | Jiangsu | 2009 | | KJ578856 | 1u |
| LN309-5 | Beef | | Liaoning | 2012 | | KJ578803 | 1u |
| LN309-9 | Beef | | Liaoning | 2012 | | KJ578804 | 1m |
| LN311-15 | Beef | | Liaoning | 2012 | | KJ578799 | 1b |
| LN311-17 | Beef | | Liaoning | 2012 | | KJ578798 | 1b |
| LN311-25 | Beef | | Liaoning | 2012 | | KJ578797 | 1b |
| LN311-27 | Beef | | Liaoning | 2012 | | KJ578796 | 1b |
| LN311-34 | Beef | | Liaoning | 2012 | | KJ578795 | 1b |
| LN313-15 | Beef | | Liaoning | 2012 | | KJ578800 | 1b |
| LN314-19 | Beef | | Liaoning | 2012 | | KJ578801 | 1b |
| LN314-21 | Beef | | Liaoning | 2012 | | KJ578802 | 1m |
| LN317-6 | Beef | | Liaoning | 2012 | | KJ578795 | 1m |
| NMG311-2 | Beef | | Inner Mongolia | 2012 | | KJ578867 | 1m |
| NMG311-20 | Beef | | Inner Mongolia | 2012 | | KJ578866 | 1u |
| NMG311-3 | Beef | | Inner Mongolia | 2012 | | KJ578865 | 1m |
| NMG312-26 | Beef | | Inner Mongolia | 2012 | | KJ578864 | 1m |
| NMG312-32 | Beef | | Inner Mongolia | 2012 | | KJ578863 | 1m |
| NMG313-1 | Beef | | Inner Mongolia | 2012 | | KJ578871 | 1m |
| NMG313-28 | Beef | | Inner Mongolia | 2012 | | KJ578870 | 1m |
| NMG313-35 | Beef | | Inner Mongolia | 2012 | | KJ578869 | 1m |
| NMG313-55 | Beef | | Inner Mongolia | 2012 | | KJ578868 | 1m |
| NMG314-22 | Beef | | Inner Mongolia | 2012 | | KJ578858 | 1m |
| NMG314-51 | Beef | | Inner Mongolia | 2012 | | KJ578860 | 1m |
| NMG314-60 | Beef | | Inner Mongolia | 2012 | | KJ578859 | 1m |
| NMG314-65 | Beef | | Inner Mongolia | 2012 | | KJ578872 | 1m |
| NMG315-1 | Beef | | Inner Mongolia | 2012 | | KJ578861 | 1m |
| NMG315-5 | Beef | | Inner Mongolia | 2012 | | KJ578862 | 1u |
| QHHY-21 | Yak | | Qinghai | 2012 | | KJ578902 | 1b |
| QHHY-22 | Yak | | Qinghai | 2012 | | KJ578903 | 1m |
| QHMY-N5 | Yak | | Qinghai | 2012 | | KJ578900 | 1b |
| QHMY-N6 | Yak | | Qinghai | 2012 | | KJ578901 | 1b |
| QHQL-126 | Yak | | Qinghai | 2012 | | KJ578880 | 1b |
| QHQL-216 | Yak | | Qinghai | 2012 | | KJ578881 | 1m |
| QHQL-219 | Yak | | Qinghai | 2012 | | KJ578890 | 1b |
| QHQL-225 | Yak | | Qinghai | 2012 | | KJ578882 | 1m |
| QHQL-252 | Yak | | Qinghai | 2012 | | KJ578884 | 1u |
| QHQL-268 | Yak | | Qinghai | 2012 | | KJ578885 | 1m |
| QHQL-271 | Yak | | Qinghai | 2012 | | KJ578891 | 1b |
| QHQL-292 | Yak | | Qinghai | 2012 | | KJ578883 | 1b |
| QHQL-295 | Yak | | Qinghai | 2012 | | KJ578886 | 1b |
| QHQL-297 | Yak | | Qinghai | 2012 | | KJ578892 | 1b |
| QHQL-299 | Yak | | Qinghai | 2012 | | KJ578893 | 1b |
| QHQL-311 | Yak | | Qinghai | 2012 | | KJ578873 | 1b |
| QHQL-313 | Yak | | Qinghai | 2012 | | KJ578874 | 1b |
| QHQL-321 | Yak | | Qinghai | 2012 | | KJ578877 | 1b |
| QHQL-328 | Yak | | Qinghai | 2012 | | KJ578875 | 1b |
| QHQL-336 | Yak | | Qinghai | 2012 | | KJ578887 | 1b |
| QHQL-337 | Yak | | Qinghai | 2012 | | KJ578876 | 1b |
| QHQL-340 | Yak | | Qinghai | 2012 | | KJ578878 | 1b |
| QHQL-345 | Yak | | Qinghai | 2012 | | KJ578879 | 1b |
| QHQL-385 | Yak | | Qinghai | 2012 | | KJ578888 | 1b |
| QHQL-405 | Yak | | Qinghai | 2012 | | KJ578889 | 1b |
| QHTJ-17 | Yak | | Qinghai | 2012 | | KJ578896 | 1b |
| QHTJ-291 | Yak | | Qinghai | 2012 | | KJ578894 | 1b |
| QHTJ-303887 | Yak | | Qinghai | 2012 | | KJ578899 | 1m |
| QHTJ-L332 | Yak | | Qinghai | 2012 | | KJ578895 | 1b |
| QHTJ-N303868 | Yak | | Qinghai | 2012 | | KJ578897 | 1b |
| QHTJ-N304810 | Yak | | Qinghai | 2012 | | KJ578898 | 1b |
| XZ-103 | Yak | | Tibet | 2010 | | KJ578916 | 1m |
| XZ-109 | Yak | | Tibet | 2010 | | KJ578917 | 1m |
| XZ-117 | Yak | | Tibet | 2010 | | KJ578904 | 1b |
| XZ-133 | Yak | | Tibet | 2010 | | KJ578906 | 1m |
| XZ-141 | Yak | | Tibet | 2010 | | KJ578907 | 1m |
| XZ-176 | Yak | | Tibet | 2010 | | KJ578905 | 1m |
| XZ-24 | Yak | | Tibet | 2010 | | KJ578918 | 1m |
| XZ-25 | Yak | | Tibet | 2010 | | KJ578908 | 1m |
| XZ-48 | Yak | | Tibet | 2010 | | KJ578909 | 1m |
| XZ-69 | Yak | | Tibet | 2010 | | KJ578910 | 1m |
| XZ-70 | Yak | | Tibet | 2010 | | KJ578911 | 1b |
| XZ-71 | Yak | | Tibet | 2010 | | KJ578912 | 1m |
| XZ-84 | Yak | | Tibet | 2010 | | KJ578913 | 1m |
| XZ-86 | Yak | | Tibet | 2010 | | KJ578914 | 1m |
| XZ-92 | Yak | | Tibet | 2010 | | KJ578915 | 1m |
| **BVDV-1 reference strains retrieved from GenBank** | | | | | | | |
| NADL | | Cattle | USA | 1963 | | AJ133739 | 1a |
| 2204–82 | | Cattle | Germany | 1982 | | AJ304377 | 1a |
| VEDEVAC | | Cattle | Hungary | 2003 | | AJ585412 | 1b |
| Osloss | | Cattle | Germany | 1965 | | M96687 | 1b |
| Manas-1 | | Cattle | China | 2006 | | EU555288 | 1b |
| AQMZ02AI21-2 | | Cattle | Australia | 2002 | | AB300687 | 1c |
| Shihezi148 | | Cattle | China | 2006 | | EU159700 | 1c |
| Manasi | | Cattle | China | 2006 | | EU159702 | 1c |
| Letuyi | | Cattle | China | 2006 | | EU159701 | 1c |
| F-Au | | Cattle | Austria | 1998 | | AF298065 | 1d |
| NCP03 | | Commercial bovine fetal sera | Japan | 2007 | | AB359927 | 1d |
| IT99-7101 | | Cattle | Italy | 1999 | | AJ318618 | 1e |
| IT99-7164 | | Cattle | Italy | 1999 | | AJ318624 | 1e |
| J-Au | | Cattle | Austria | 1998 | | AF298067 | 1f |
| W-Au | | Cattle | Austria | 1998 | | AF298073 | 1f |
| A-Au | | Cattle | Austria | 1998 | | AF298064 | 1g |
| L-Au | | Cattle | Austria | 1998 | | AF298069 | 1g |
| G-Au | | Cattle | Austria | 1998 | | AF298066 | 1h |
| 23–15 | | Cattle | UK | 1997 | | AF298059 | 1i |
| Deer | | Deer | Japan | 2000 | | AB040132 | 1j |
| KS86-1ncp | | Cattle | Japan | 2002 | | AB078950 | 1j |
| Rebe | | - | Switzerland | 2000 | | AF299317 | 1k |
| Suwa | | - | Switzerland | 1999 | | AF117699 | 1k |
| ZM-95 | | Pig | China | 1995 | | AF526381 | 1m |
| 06z71 | | Cloned virus propagated in cells | South Korea | 2006 | | DQ973181 | 1n |
| Shitara0206 | | Cattle | Japan | 2006 | | AB359930 | 1n |
| SoCP-75 | | Cattle | Japan | 1975 | | AB359929 | 1n |
| AQGN96BI5 | | Cattle | China | 1996 | | AB300691 | 1o |
| IS25CP01 | | Cattle | Japan | 2001 | | AB359931 | 1o |
| BJ0702 | | Cattle | China | 2007 | | GU120248 | 1p |
| BJ0703 | | Cattle | China | 2007 | | GU120249 | 1p |
| 11N36 | | Cattle | China | 2011 | | JX437156 | 1q |
| M31182 | | Yak | China | 2010 | | JQ799141 | 1u |
| UM/136/08 | | Cattle | Italy |  | | LM994673 | 1s |
| SI/207/12 | | Cattle | Italy |  | | LM994674 | 1t |
| **BVDV-2 reference strains retrieved from GenBank** | | | | | | | |
| AQHY93BI22 | | Cattle | USA | 1993 | AB300664 | | 2 |
| XJ-04 | | Cattle | China | 2004 | FJ527854 | | 2 |
|  | |  |  |  |  | |  |
